# Pancreatic amylase activity and development of the gastrointestinal tract in C57BL/6J mice before and after weaning

**DOI:** 10.1038/s41598-026-44974-8

**Published:** 2026-03-26

**Authors:** Annick Ernst, Linda F. Böswald

**Affiliations:** Core Facility Animal Models, Biomedical Center, Medical Faculty, Ludwig-Maximilians- Universität München, Großhaderner Str. 9, 82152 Planegg-Martinsried, Germany

**Keywords:** Enzymatic activity, Digestion, Starch, Caecum development, Pancreas, Gastroenterology, Physiology, Zoology

## Abstract

In mammals, the period of weaning represents the change from milk consumption to a solid, species-specific diet. This is associated with adaptations of the gastrointestinal tract, including the digestive enzymes. This study aimed to investigate the amylase activity in pancreas tissue and small intestinal content before, at and after weaning in C57BL/6J mice, in addition to determining blood glucose levels, body weight, organ weights and the length of the small intestine and colon. In total, 59 mice were sacrificed at the ages of 12 d, 3, 4, 5, 6, 8 and 10 weeks, to obtain these parameters. Weaning had been set to take place at 21 d. Age groups were statistically compared with one-way analyses of variances (*α* = 0.05). Body weight followed a non-linear function of age. Relative liver weight increased abruptly from 3 to 4 weeks of age, while relative spleen weight decreased from 4 to 8 weeks of age. Relative pancreas weight increased significantly until 6 weeks of age. Pancreatic amylase activity significantly increased from 3 to 4 weeks of age, corresponding to increasing intake of solid diet at weaning. Amylase activity in small intestinal content did not follow this pattern, possibly because of a non-representative nature of the samples. Further studies are warranted to test for the effect of weaning age, diet and genetic background on amylase activity in mice.

**Supplementary Information:**

The online version contains supplementary material available at 10.1038/s41598-026-44974-8.

## Introduction

Carbohydrates, and specifically starch, are a major source of energy in animal feed. In the digestive tract of monogastric species, the polysaccharide starch is digested mainly by pancreatic α-amylase. This enzyme is a hydrolase cleaving the α,1–4 glycosidic bonds in the amylose or amylopectin chains within the starch molecule. This renders smaller carbohydrates like maltotriose, maltose, glucose and limit dextrins for further degradation or absorption, respectively^1,2^.

In pet and farm animal species, like dogs^3,4^, cats^5^, horses^6^, and pigs^7,8^, amylase levels and their increase with age and diet have been studied in detail. There is also some data in rats^9–12^, even though this has been obtained under husbandry and feeding conditions not directly comparable to the modern standards in laboratory animal science. The activity of pancreatic amylase in adult animals shows clear species differences, which can be associated with the diet type. Omnivores with higher amounts of carbohydrates, and starch, in their natural diets, tend to have the highest pancreatic amylase activities^13,14^. Herbivores feeding on “leafy material”, i.e. forage without considerable starch content, have considerably less amylase^6,15–19^. Strict carnivores like cats have negligible amylase activity which cannot be stimulated by increasing dietary starch^5^, whereas dogs have adapted to man-made diets containing starch during domestication, having higher and inducible amylase activity^3,4^. In a previous study on species differences between laboratory rodents, we compared the pancreatic amylase activity between C57BL/6J mice, Sprague Dawley rats, and hamsters. The findings showed that mice and rats had significantly higher levels than hamsters, possibly related to the complex stomach of the hamster enabling microbial “foregut fermentation” and reducing the need for small intestinal starch degradation^20^.

There is also a distinct effect of age on amylase activity. After birth, mammalian offspring is consuming mother´s milk in the first period of life. The digestive system is adapted to this in several aspects. Lactose is the main carbohydrate in milk, and the neonate have high activity levels of the enzyme lactase to digest this. During maturation and increasing ingestion of solid feed, lactase activity decreases^21,22^ because lactose is not usually part of “natural” diets^23^. With the intake of solid feed, starch will become more important as source of energy. Correspondingly, the activity of the pancreatic enzyme amylase increases^22,23^. This, however, has not yet been studied in laboratory rodents.

Knowledge about the enzymatic capacity for digestion of solid feed is required to refine the weaning age and possible pre-weaning support to make the transition to solely solid feed easier for the offspring. In addition, detailed data on the digestive physiology of laboratory animals is essential to understand the organism that is used as research model in many disciplines. In terms of the 3Rs^24^, Refinement requires knowledge about the model organism to design experiments in the best-possible was and also interpret data under consideration of all species-specific peculiarities.

The aim of the present study was to investigate the activity of pancreatic amylase in C57BL/6J mice before, at and after weaning. In addition, amylase activity in the small intestinal content and several parameters regarding the growth of organs and development of the gastrointestinal tract were determined.

## Materials and methods

### Animals

The project was carried out in accordance with the appropriate European and German animal welfare legislations (5.1–231 5682/LMU/BMC/ project reference number CAM 2025-019; sacrifice as approved under § 4 German Animal Welfare Act (*Tierschutzgesetz*)). The project was approved by the animal welfare body of the Core Facility Animal Models, BMC, LMU, and all conditions regarding the use of animals are reported in accordance with the ARRIVE guidelines.

We used 59 C57BL/6J mice bred in our facility (Core Facility Animal Models, Biomedical Center, LMU München, Germany) and housed under specified-pathogen-free conditions in individually ventilated cages (Type II long, Tecniplast S.p.A., Buggugiate, Italy). The husbandry rooms were kept at defined climate settings (room temperature 20–22 °C, relative humidity 45–55%, light cycle 12 h light:12 h dark, room air exchange 11x per hour) in individually ventilated cages (HEPA-filtered air flow). The cages were fitted with aspen bedding material (LAS bedding PG3, Altromin Spezialfutter GmbH Co., Lage, Germany), a red corner house (Tecniplast) and enrichment (5 × 5 cm nestlet, Datesand, UK; plastic tunnel). Room air was exchanged 11 times per hour and filtered with HEPA-systems. The hygiene monitoring adhered to the FELASA-14 recommendations (every three months). All mice in the facility were fed *ad libitum* with the pelleted diet Altromin 1314P (irradiated for sterilization), so that this diet was available in the breeding cages of the mice bred specifically for this study. Demineralized, filtered water was available at all times.

### Study design

The mice were bred specifically for the study and for each litter, the date of sacrifice was set at a defined age: 12 days (*n* = 7), 3 weeks (*n* = 8), 4 weeks (*n* = 9), 5 weeks (*n* = 11), 6 weeks (*n* = 8), 8 weeks (*n* = 8), and 10 weeks (*n* = 8). Weaning was set at 21 days of age and before weaning, no feed was put on the cage floor (only available on the cage grid). Up to weaning, the litters were housed with their mother in one cage. At weaning, the litter was separated from the mother and grouped according to sex (maximum of five mice of the same sex in one cage). One or two litters were used for each age point. The mice were sacrificed via cervical dislocation and dissected immediately. Cardiac blood was taken for measuring blood glucose (FreeStyle Lite glucometer, Abbott, Canada) in duplicate to calculate the mean per animal. The complete pancreas was removed to weigh it and use it for analysis of amylase activity. In addition, small intestinal content from a defined site of approximately 2 cm in the middle of the small intestine was sampled for amylase activity determination. The length of the small intestine and the colon was measured with a ruler. Several organs were removed *in toto* and weighed (heart, stomach, liver, spleen, caecum, kidneys).

Amylase activity was determined with the Phadebas^®^ kit (Phadebas AB, Kristianstad, Sweden). Initially, the pancreas and small intestinal content, respectively, were diluted 1:1000 in bovine serum albumin buffer as described in the protocol. After homogenisation with an Ultra-Turrax^®^ T10 basic (IKA^®^ Werke GMbH & Co. KG, Staufen, Germany), 200 µL of the sample homogenate was added to demineralized water and incubated with the Phadebas^®^ tablet for 5 min (as instructed for a very high expected amylase activity) in a water bath at 37 °C. After stopping the reaction by adding 1 mL of 5 Mol NaOH, the samples were centrifuged and filtrated. The colour intensity of the supernatant was measured at 620 nm (VersaMax Microplate Reader, Molecular Devices LLC., San Jose, CA, USA) and translated into enzyme activity units with the standard curve of the Phadebas^®^ tablets.

### Statistics

GraphPad Prism^®^ version 10.6.1 was used (Graphpad Software, San Diego, CA, USA) was used for statistical analysis. The level of statistical significance was set to *α* = 0.05. With two-way analyses of variance (ANOVA), the influence of age and sex (independent variables) on the dependent variable (body and organ weights, amylase activity, respectively) was determined. If only one factor was tested, a one-way ANOVA was conducted. To test for correlations between parameters, linear regressions were calculated. Box plots were created to illustrate data distribution, with the boxes representing the middle 50% of data, the horizonal line in the box the median, and the whiskers extending to the minimum and maximum of data.

## Results

**Fig. 1:**
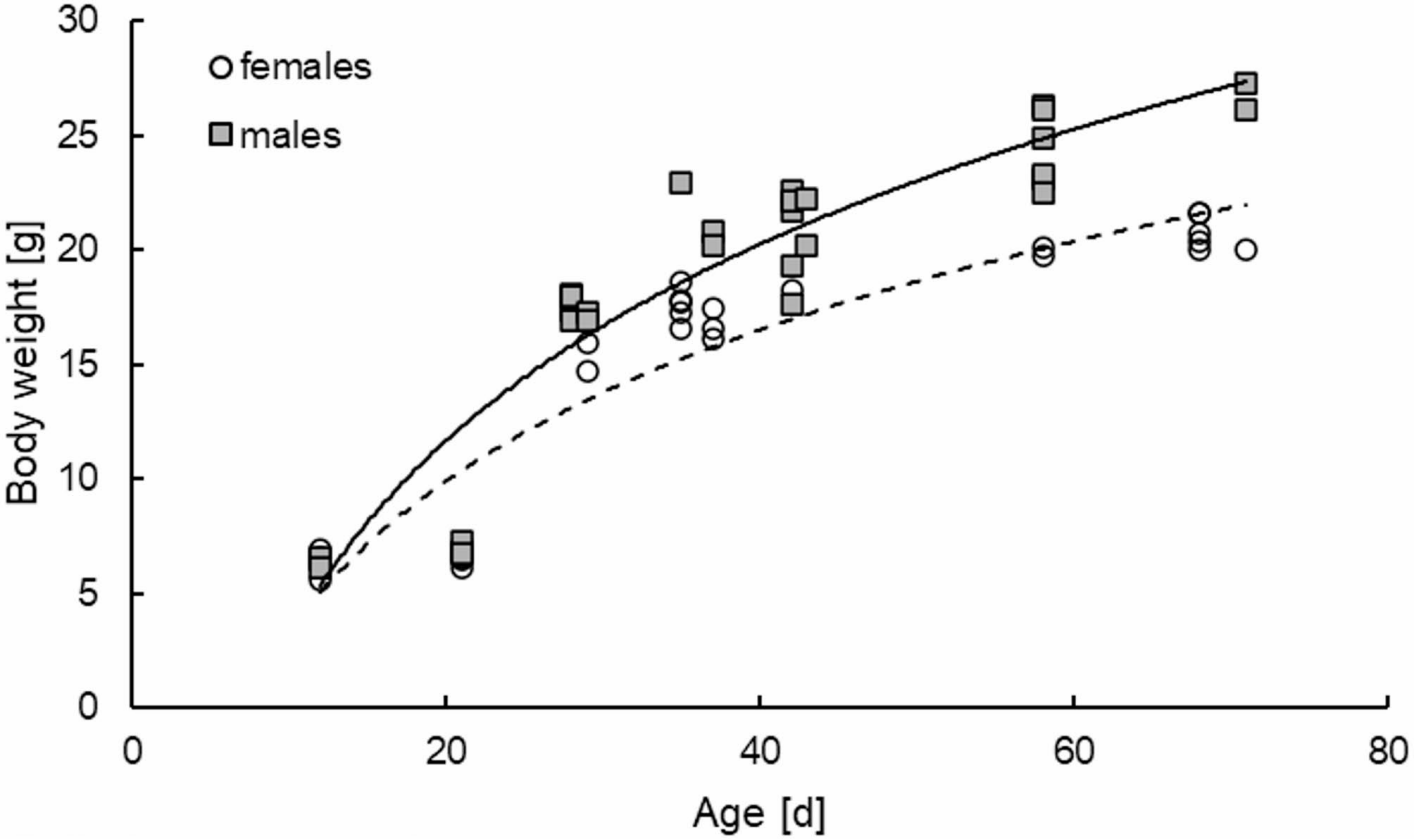
Body weight as function of age, plotted separately for males (grey rectangles; *n* = 31; y = 12.4ln(x) – 25.48; R² = 0.87) and females (white circles; *n* = 28; y = 9.54ln(x) – 18.68; R² = 0.86).

Body weight was significantly influenced by age (F(6,45) = 307.5; *p* < 0.0001, 82.79% of total variation) and sex (F(1,45) = 94.81; *p* < 0.0001, 4.25% of total variation) (Table 1, Supplementary Figure S1A). Age and body weight were connected in logarithmic function, demonstrating that the sex differences becoming more pronounced over time (Fig. 1).

The relative organ weights are given in Table 1, the absolute values in Supplementary Table S1. Relative heart weight was significantly influenced by age (F(6,45) = 2.826; *p* < 0.05, 24.05% of total variation) but not sex (F(1,45) = 0.1595; *p* = 0.692). The same was true for relative weight of the left kidney (factor age: F(6,45) = 3.770; *p* < 0.01, 29.18% of total variation; factor sex: F(1,45) = 0.01066; *p* = 0.918), the liver (factor age: F(6,45) = 33.20; *p* < 0.0001, 71.43% of total variation; factor sex: F(1,45) = 2.58; *p* = 0.118), the spleen (factor age: F(6,45) = 3.864; *p* < 0.01, 29.11% of total variation; factor sex: F(1,45) = 0.2425; *p* = 0.625), the pancreas (factor age: F(6,45) = 11.22; *p* < 0.0001, 53.83% of total variation; factor sex: F(1,45) < 0.01 ; *p* = 0.995). The relative weight of the right kidney was not significantly affected by age (F(6,45) = 0.7058; *p* = 0.647) or sex (F(1,45) = 1.153; *p* = 0.289).

Stomach and caecum were both weight with their content and set in relation to body weight. Age had a significant effect on relative stomach weight (F(6,45) = 6.758; *p* < 0.0001, 34.10% of total variation) and on relative caecum weight (F(6,45) = 39.53; *p* < 0.0001, 64.39% of total variation), while sex did not significantly affect both (stomach: *p* = 0.609; caecum: *p* = 0.260). The caecum was very small and visibly empty in the 12 days-old mice.

Blood glucose levels ranged from 103.5 to 212 mg/dL and were significantly influenced by sex with males having higher values (F(1,45) = 7.40; *p* < 0.05), but not influenced by age (F(6,45) = 1.997; *p* = 0.106).

**Fig. 2:**
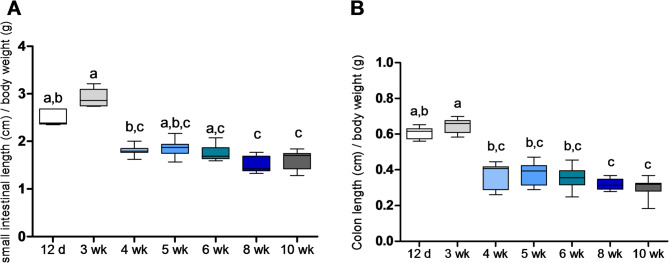
Relative length of the (**A**) small intestine and (**B**) colon at the different ages. The boxes represent the 25–75% quartiles of data with the median as a horizontal line and the whiskers indicate the rest of data. Boxes with differing letters above them differ significantly (*p* < 0.05).

Both small intestine and colon increased in length from 12 days until 5 weeks in similar fashion (Fig. 2). Age had a significant effect on small intestinal length (F(6,45) = 162.8; *p* < 0.0001, 82.71% of total variation) and on colon length (F(6,45) = 13.78; *p* < 0.0001, 53.41% of total variation), while sex did not significantly affect both (small intestine: *p* = 0.119; colon: *p* = 0.053, potential trend). To correct for body weight, the length of the intestinal parts (cm) was related to body weight (g), showing significant differences according to age (for both small intestine and colon: *p* < 0.0001; Fig. 2).

**Table 1:** Overview of body weights, blood glucose levels and organ weights and lengths of the C57Bl/6J mice at different ages.

		12 d	3 weeks	4 weeks	5 weeks	6 weeks	8 weeks	10 weeks
		*n* = 7	*n* = 8	*n* = 9	*n* = 11	*n* = 8	*n* = 8	*n* = 8
Body weight	*g*	6.37 ± 0.42 [5.60; 6.90]	6.68 ± 0.33 [6.15; 7.30]	17.02 ± 1.07 [14.75; 18.05]	18.38 ± 2.12 [16.15; 22.95]	20.53 ± 2.16 [17.60; 22.60]	23.28 ± 2.47 [19.80; 26.35]	22.23 ± 2.86 [20.05; 27.30]
Heart weight	*% BW*	0.69 ± 0.13 [0.52; 0.94]	0.81 ± 0.14 [0.68; 1.09]	0.69 ± 0.09 [0.58; 0.89]	0.60 ± 0.10 [0.49; 0.87]	0.72 ± 0.11 [0.54; 0.87]	0.68 ± 0.05 [0.60; 0.76]	0.66 ± 0.07 [0.54; 0.73]
Liver weight	*% BW*	3.20 ± 0.68 [2.66; 4.66]	3.69 ± 0.20 [3.35; 3.87]	6.70 ± 0.52 [5.93; 7.53]	6.51 ± 0.48 [05.49; 7.77]	6.42 ± 0.83 [4.63; 7.11]	5.76 ± 0.74 [4.23; 6.38]	5.51 ± 0.0.79 [3.71; 6.37]
Spleen weight	*% BW*	0.41 ± 0.10 [0.27; 0.58]	0.44 ± 0.07 [0.36; 0.59]	0.38 ± 0.06 [0.31; 0.48]	0.37 ± 0.05 [0.31; 0.45]	0.33 ± 0.04 [0.27; 0.40]	0.33^b^ ± 0.06 [0.26; 0.44]	0.38 ± 0.06 [0.29; 0.50]
Left kidney weight	*% BW*	0.66 ± 0.06 [0.61; 0.77]	0.81 ± 0.10 [0.67; 1.00]	0.69 ± 0.05 [0.57; 0.72]	0.65 ± 0.04 [0.59; 0.70]	0.71 ± 0.05 [0.63; 0.80]	0.74 ± 0.07 [0.66; 0.90]	0.72 ± 0.10 [0.58; 0.91]
Right kidney weight	*% BW*	0.64 ± 0.16 [0.38; 0.88]	0.80 ± 0.05 [0.74; 0.86]*	0.70 ± 0.09 [0.62; 0.87]	0.70 ± 0.11 [0.50; 0.89]	0.76 ± 0.06 [0.67; 0.84]	0.75 ± 0.07 [0.69; 0.89]	0.73 ± 0.05 [0.63; 0.81]
Pancreas weight	*% BW*	0.31 ± 0.14 [0.20; 0.62]	0.44 ± 0.23 [0.20; 0.81]	0.52 ± 0.05 [0.43; 0.57]	0.67 ± 0.06 [0.60; 0.77]	0.74 ± 0.18 [0.55; 1.14]	0.76 ± 0.10 [0.59; 0.90]	0.81 ± 0.13 [0.68; 1.10]
Stomach weight	*% BW*	1.94 ± 0.31 [1.46; 2.31]	1.72 ± 0.70 [1.05; 3.21]	2.92 ± 0.56 [1.76; 3.99]	1.92 ± 0.53 [1.39; 3.31]	1.80 ± 0.67 [0.92; 2.85]	1.17 ± 0.22 [0.84; 1.54]	1.62 ± 0.55 [0.74; 2.62]
Caecum weight	*% BW*	0.27 ± 0.19 [0.01; 0.51]	1.13 ± 0.27 [0.77; 1.60]	3.15 ± 0.82 [2.34; 4.86]	2.95 ± 0.39 [2.27; 3.36]	2.67 ± 0.38 [2.06; 3.38]	2.29 ± 0.44 [1.61; 3.02]	3.25 ± 0.69 [2.21; 4.35]
Small intestinal length	*cm*	15.79 ± 1.07 [14.5; 17.5]	19.50 ± 1.51 [17; 22]	30.78 ± 2.74 [26.5; 34]	33.91 ± 2.25 [31; 38]	35.56 ± 1.99 [31.5; 38]	34.44 ± 1.35 [32; 36]	35.69 ± 1.16 [34; 37.5]
Colon length	*cm*	3.86 ± 0.35 [3.5; 4.5]	4.31 ± 0.26 [4; 4.5]	6.28 ± 1.35 [4.5; 8]	7.07 ± 1.27 [5; 9]	7.19 ± 0.92 [5; 9]	7.44 ± 1.12 [5.5; 9]	6.56 ± 0.82 [5.0; 7.5]

		*n* = 6	*n* = 8	*n* = 4	*n* = 6	*n* = 6	*n* = 5	*n* = 3
Blood glucose	*mg/dL*	126.64 ± 20.94 [106; 153]	153.10 ± 31.65 [103.5; 212]	133.63 ± 22.31 [115; 166]	143.75 ± 24.12 [109.5; 172]	151.53 ± 22.17 [123.5; 185]	129.40 ± 19.60 [111.5; 159.5]	142.08 ± 20.22 [128; 165.25]

Data is presented as mean ± standard deviation [minimum; maximum] per age group and parameter. BW = body weight. Stomach and caecum were weighed with content.

*n = 7

## Amylase activity

Age had a significant effect on pancreatic amylase activity given as Units per gram tissue (F(6,44) = 3.397; *p* < 0.01, 24.34% of total variation), while sex did not (F(1,44) = 1.322; *p* = 0.256). The same was true for amylase activity per pancreas per animal (F(6,44) = 27.00; *p* < 0.0001, 68.32% of total variation), while sex did not (F(1,44) = 0.007; *p* = 0.936).

Amylase activity in the small intestinal content could not be determined in the 12 days-old mice because of the negligible quantity of small intestinal content. Neither age (*p* = 0.899) nor sex (*p* = 0.886) had a significant impact on amylase activity measured in the small intestinal content (Table 2, Supplementary Figure S2).

**Table 2:** Amylase activity in pancreatic tissue and small intestinal content.

		12 d	3 weeks	4 weeks	5 weeks	6 weeks	8 weeks	10 weeks
		*n* = 7	*n* = 8	*n* = 9	*n* = 11	*n* = 8	*n* = 8	*n* = 8
Pancreas amylase	*U/g wet weight*	1979 ± 687 [1004; 2857]	3398 ± 1409 [1497; 5628]	4083 ± 1107 [2451; 5163]	3783 ± 1024 [1965; 4920]	4059 ± 729 [2987; 5234]	3995 ± 754 [2861; 4872]	3318 ± 1220 [1551; 4872]
Pancreas amylase	*U/pancreas*	39 ± 14 [16; 56]	92 ± 47 [47; 195]	367 ± 124 [184; 512]	462 ± 129 [224; 622]	608 ± 120 [332; 843]	693 ± 106 [543; 857]	572 ± 169 [347; 730]

			*n* = 8	*n* = 4	*n* = 6	*n* = 6	*n* = 5	*n* = 3
Small intestinal content amylase	*U/g wet weight*	n.a.	1836 ± 1188 [870; 3770]	1558 ± 221 [1314; 1836]	2375 ± 457 [1682; 2943]	1784 ± 392 [1254; 2120]	1920 ± 1213 [477; 3475]	2197 ± 814 [1329; 2943]

Data is presented as mean ± standard deviation [minimum; maximum] per age group and parameter. N.a. = not analysed; U = units.

## Discussion

In the present study, we used C57BL/6J mice to study the development of gastrointestinal parameters around weaning. This strain was chosen because it is one of the most commonly used wild type strains or as background for genetically modified mice. Potential genetical differences in other strains of mice cannot be excluded with the present data but need to be investigated specifically.

One or two litters were available per age group, with both male and female mice as born in these litters. We did not expect to see sex differences in amylase activity, which was the main parameter of the study, so that we decided not to breed for more mice, which also would generate surplus animals. This would not meet the “Reduce” principle of the 3Rs^24^. Supporting this, no sex differences in pancreatic amylase activity were detected in weaning piglets^25^. The main difference between males and females observed in this study was in body weight and the correlation between body weight and age (Fig. 1), which is to be expected as a sexual dimorphism in body weight development and metabolism has been described in C57BL/6 mice^26,27^. Analysing all further parameters for potential effects of age and sex in a two-way ANOVA did not show any influence of sex, so that the tabular and visual presentation of data grouped according to age is justified.

Overall, body weight in male and female mice increased in a non-linear function of age, which is known similarly from other species, such as dogs, cats, sheep, cattle, pigs, and rats^28–35^. For mice of other strains, multi-phase growth curves have been described with a good fit, indicating that there are different phases of growth, i.e. different intensities of weight gain^36^. The highest growth rate seems to be reached from 1 to 2 months of age, after which the slope of the growth curve lowers^37^.

For standardization purposes, the weaning age was set at 21 days for all mice. Solid feed was available only on the cage grid, i.e. the young mice had to be able to climb in order to reach and consume it. In the common practice, mice are weaned between 18 and 28 days of age^38^, so that the age chosen in this study is well within this range. In this period, a difference of days can have a high impact on physiology, so that a variation of weaning age might have an influence on the outcome, as discussed below for the amylase activity.

The organ weight data can be valuable for the creation of reference values to identify abnormalities in mice with disease or genetic modifications. Concurrent with the increase in body weight, the relative weights of most organs showed an increase and a sort of “leap” from 3 to 4 weeks. In weaning pigs, relative organ weights were also found to increase with age being the major determinant. A possible explanation is the increase in nutrient supply from the solid feed, being available for tissue growth^25^. In the mice from the present study, the relative liver weight showed an abrupt and significant rise at 4 weeks from 3 to 4% of body weight to around 6% of body weight. This might be related to metabolic changes after weaning, when solid feed based on carbohydrates becomes the only source of energy. After weaning, the lipogenic capacity of the liver and the hepatic glycogen storage increase^39^. This hypothesis might be tested by analysing the livers for gross energy, lipid and carbohydrate content, which was not performed in the present study.

The relative spleen weight increased to weaning and decreased significantly between 6 and 10 weeks. This might be cautiously interpreted as indication of reduced haematopoiesis, one of the spleen´s major functions^40^, or alterations in the immune cell counts during maturation of the mice post-weaning. Spleen weight can correlate with the cellular immune response^41^. In rats, absolute spleen weight and concurrently spleen cell counts, increased until the age of 9 weeks, then decreased slightly at 11 weeks of age^42^. When the spleen weights given in the cited paper^42^ are calculated as percent of body weight, they show a marked decrease from 5 weeks (spleen weight 0.35% BW) to 11 weeks (spleen weight 0.18% BW). The relative spleen weight in the 5-week-old rats is surprisingly similar to the percentage determined in the mice from the present study (0.37 ± 0.05% BW). To investigate the background of the observed change in relative spleen weight, further parameters like cell counts and histology would be necessary.

Stomach weight did differ significantly between the age groups and showed considerable variation. Since the stomach was weighed with its content, feed intake is the major determinant of the weight. We did not control feed intake before sacrifice, so that the absence of a systematic pattern in this parameter is likely due to the stomach filling dominating the weight of the organ including its content. The only finding that might be of relevance in this regard is the significantly higher stomach weight at 4 weeks of age (Supplementary Fig. S1H). This timepoint is 1 week after weaning, so is might very tentatively be interpreted as indication of an increase in stomach filling on the solid diet.

Small intestinal length increased significantly with age until 5 weeks of age, after which there were no significant differences up to 10 weeks. With a similar rate, colon length increased significantly from until 4 weeks of age. For the “house mouse of a laboratory strain” with 43 g mean BW, a colon length of 8.5 cm has been reported^43^. This reference stems from zoological research and does not report details on housing or feeding conditions, so that a direct comparison to mice under specified pathogen-free conditions fed standardized pelleted diets may be limited.

Mice are hindgut fermenters with an enlarged caecum which serves as a fermentation chamber for pre-caecally indigestible particles, especially fibre and starch^22,44^. The development of the caecum has not been studied in mice to the authors´ knowledge, so that the relative caecum weight data obtained in this study can give first insights. The relative weight of the filled caecum (% BW) can be an easy to determine indicator of fermentative activity, for example in mice fed high-fibre diets^45^ and in rats fed different types of starch^46^. In the 12-days-old mice, the caecum was a very small appendage at the transition of small to large intestine. Macroscopically, it seemed to be nearly empty or filled only with small amounts of clear fluid. The relative caecum weight increased significantly from 12 d to 3 weeks, consistent with the start of ingestion of solid feed by the young mice before weaning. At 3 weeks of age, the caecum content visibly contained feed particles. The relative caecum weight at 4 weeks, when the mice had been solely consuming solid feed for at least a week, was significantly higher than at 3 weeks. It can be assumed that the start of the ingestion of solid feed leads to changes in the microbial activity^47,48^, supported by the increase in the relative weight of the filled organ. In the weaned mice, the relative caecum weight was comparable to that determined in adult rats^46^ but higher than that of young, weaned rabbits^49^.

In the present study, blood glucose values did not differ significantly between the age groups and showed no systematic pattern in distribution. This is most likely due to the non-standardization of postprandial time of measuring blood glucose. The mice had *ad libitum* access to the diet right until the sacrifice, but the last actual intake of feed and the amount of this meal could not be controlled. Since feed intake is the major determinant of blood glucose levels, the sampling setup explains the variability. However, fasting small rodents can have marked effects in itself^50,51^ and mice cannot be meal-fed like dogs or pigs, so it is difficult to set up a standardized procedure with spontaneous, voluntary feed consumption. Glucose tolerance tests with intravenous or orogastric application of a defined glucose bolus would render controlled results but are rather invasive and were not the aim of this study. Apart from the variation, the blood glucose values measured in this study were in the range reported as reference values for C57BL/6J mice aged 3–7 months (fasting or fed state not reported in the paper)^37^.

To the authors´ knowledge, the amylase activity in mice has not been investigated in the peri-weaning period. In mammalian species, it is known that during the suckling period, the offspring has digestive enzymes adapted to mother´s milk and that the increasing intake of non-milk diet around the time of weaning induces the synthesis and activity of enzymes to digest this diet. It is assumed that the beginning and then sole intake of solid feed induces pancreatic maturation^52,53^. The relative pancreas weight in the mice increased up to 5–6 weeks of age, as shown similarly in piglets^25^. The amylase activity was investigated in young rats^9,11,12^, dogs^3,54^, pigs^55^, as examples of other monogastric animals, so that the aim of the present study was to obtain data from a commonly used laboratory mouse strain. Pancreatic amylase activity was measured by homogenizing the complete pancreas tissue for analysis. This method was successfully used in laboratory rodents in a previous study, comparing the enzymatic activity between young adult mice, rats and hamsters^20^. The age points were chosen to start at an age (12 d) when the young mice solely consume mother´s milk. Visual evaluation of the gastrointestinal tract and its content confirmed that the 12-day-old mice had not consumed any solid feed. Their stomach was filled with milk, and the intestinal content was light in colour and had a liquid to viscous consistency.

As expected, at this age, pancreatic amylase activity had the lowest values. From empirical data, mice start to show interest in solid feed from 15 d on, and the actual intake of solid feed will increase up to weaning, after which the complete energy intake consists of the solid feed. The steep increase in pancreatic amylase activity from 12 d to 3 weeks of age is concurrent to this increasing consumption of starch-containing solid feed. Since the pancreas itself also increased in weight, the calculation of total amylase activity units per animal by multiplying the units per gram pancreas weight with the pancreas weight gave the best overview of the development of the enzyme peri-weaning (Supplementary Figure S2-B). After weaning, the pancreatic amylase activity still increased until 8 weeks, which might indicate further adaptation to the carbohydrate-based laboratory diet. The dietary starch content was constant, so that the age and/or the increased intake of the diet seem to be the determinants of the increase in amylase activity. Potentially, variations in dietary starch content in post-weaning mice might induce alterations in amylase activity, as observed for example in dogs and rats^3,12^. Weaning age could also influence the time and slope of the increase in amylase activity. For mice, it has been reported that “naturalistic” weaning starts around 14–17 d postnatal, ending around 23 d, or in some cases extend up to 35 d, but is ended abruptly by separating mother and offspring between 12 and 25 d in breeding or research facilities^56^. For this study, we chose 21 d as weaning age for all litters to ensure that the pups would all be of adequate size and maturity to separate them, which might not be the case for all individuals before < 20 d. Weaning age influences feeding frequency and meal size in piglets^57,58^. The availability of mother´s milk has been shown to decrease the motivation for voluntary consumption of solid feed in calves and piglets^59,60^, which might also be the case in mice. Since feed intake and feed composition influences amylase activity, there might be differences in the peri-weaning increase (onset, slope of increase) with different weaning ages in mice.

Small intestinal amylase activity was lower than that measured directly from pancreatic tissue, as reported previously in laboratory rodents^20^. The activity values showed a high variation (Fig. 2C), and no systematic differences between the age groups. Possibly, this is related to the abovementioned lack of standardization of the last postmortem feed intake, since feed intake will influence the fill of the small intestine and induce secretion of pancreatic juice^61^. In addition, the amount of small intestinal content sampled might not have been representative for the total content, even though the sample was taken at a defined location.

## Conclusion

In summary, the study investigated body and organ weight as well as pancreatic amylase activity in peri-weaning C57BL/6J mice. Body weight increased in a logarithmic function of age, with differences between male and female mice. The relative organ weight increased with age, with most pronounced changes until 4–5 weeks of age. As known from other mammalian species, pancreatic amylase activity increased significantly with age and beginning ingestion of solid feed. Correspondingly, the small intestine and colon expanded in length during this period, and the caecum increased in size and weight. More in-depth studies on the peri-weaning development of the murine digestive physiology and metabolism are warranted to fully understand the model organisms that researchers of all disciplines are working with.

## Supplementary Information

Below is the link to the electronic supplementary material.

Supplementary Material 1

## Data Availability

All relevant data is reported in the manuscript. Further individual data can be obtained from the authors upon reasonable request.
