# Supplementary material for: Pancreatic amylase activity and development of the gastrointestinal tract in C57BL/6J mice before and after weaning
Source: Sci Rep. 2026 Mar 26;16:10502. doi: 10.1038/s41598-026-44974-8 (PMC13031689; doi:10.1038/s41598-026-44974-8)
Supplement: Supplementary file 1 — Supplementary Material 1 [file 41598_2026_44974_MOESM1_ESM.docx]

**Supplementary Figure S1.** Box plots of the parameters A) body weight, relative weights of B) heart, C) liver, D) spleen, E) left kidney, F) right kidney, G) pancreas, H) stomach, J) caecum, and K) blood glucose. The boxes represent the 25 – 75 % quartiles of data with the median as a horizontal line and the whiskers indicate the rest of data. Boxes with differing letters above them differ significantly (*p* < 0.05) when analysed for age groups.

**Supplementary Figure S2**. Activity of amylase A) from the pancreatic tissue, expressed in units per gram of pancreas, B) from the pancreatic tissue, expressed in units per pancreas, and C) from the small intestinal content (units per gram of intestinal content). The boxes represent the 25 – 75 % quartiles of data with the median as a horizontal line and the whiskers indicate the rest of data. Boxes with differing letters above them differ significantly (p < 0.05).

**Supplementary Table S1.** Absolute organ weights of the C57Bl/6J mice at different ages.

|  | | **12 d** | **3 weeks** | **4 weeks** | **5 weeks** | **6 weeks** | **8 weeks** | **10 weeks** |
| --- | --- | --- | --- | --- | --- | --- | --- | --- |
|  |  | *n* = 7 | *n* = 8 | *n* = 9 | *n* = 11 | *n* = 8 | *n* = 8 | *n* = 8 |
| Heart weight | *g* | 0.04 ± 0.01  [0.03; 0.06] | 0.05 ± 0.01 [0.05; 0.07] | 0.12 ± 0.02 [0.09; 0.16] | 0.11 ± 0.02 [0.09; 0.14] | 0.15 ± 0.03 [0.12; 0.19] | 0.16 ± 0.02 [0.13; 0.19] | 0.15 ± 0.02 [0.12; 0.20] |
| Liver weight | *g* | 0.2 ± 0.05  [0.15; 0.30] | 0.25 ± 0.02 [0.21; 0.28] | 1.14 ± 0.13 [0.94; 1.29] | 1.20 ± 0.26 [0.97; 1.65] | 1.33 ± 0.25 [0.82; 1.58] | 1.34 ± 0.25 [1.00; 1.68] | 1.23 ± 0.23 [0.76; 1.50] |
| Spleen weight | *g* | 0.03 ± 0.01 [0.02; 0.04] | 0.03 ± 0.01 [0.02; 0.04] | 0.07 ± 0.01 [0.06; 0.08] | 0.07 ± 0.01 [0.06; 0.08] | 0.07 ± 0.01 [0.06; 0.08] | 0.08 ± 0.02 [0.06; 0.11] | 0.08 ± 0.01 [0.07; 0.10] |
| Left kidney weight | *g* | 0.04 ± 0.01 [0.04; 0.05] | 0.05 ± 0.01 [0.05; 0.07] | 0.11 ± 0.01 [0.10; 0.12] | 0.12 ± 0.02 [0.10; 0.16] | 0.15 ± 0.02 [0.13; 0.17] | 0.17 ± 0.03 [0.15; 0.22] | 0.16 ± 0.03 [0.13; 0.21] |
| Right kidney weight | *g* | 0.04 ± 0.01  [0.02; 0.06] | 0.05 ± 0.004  [0.05; 0.06]* | 0.12 ± 0.01 [0.11; 0.13] | 0.13 ± 0.02 [0.08; 0.15] | 0.16 ± 0.02 [0.13; 0.18] | 0.17 ± 0.03 [0.14; 0.22] | 0.16 ± 0.03 [0.13; 0.20] |
| Pancreas weight | *g* | 0.02 ± 0.01  [0.01; 0.04] | 0.03 ± 0.01 [0.01; 0.05] | 0.09 ± 0.01 [0.07; 0.10] | 0.12 ± 0.01  [0.11; 0.15] | 0.15 ± 0.02 [0.11; 0.20] | 0.18 ± 0.03 [0.14; 0.21] | 0.18 ± 0.04 [0.14; 0.23] |
| Stomach weight | *g* | 0.12 ± 0.02  [0.10; 0.15] | 0.12 ± 0.05 [0.07; 0.22] | 0.50 ± 0.14 [0.30; 0.68] | 0.36 ± 0.15 [0.26; 0.76] | 0.36 ± 0.11 [0.20; 0.57] | 0.28 ± 0.07 [0.19; 0.38] | 0.35 ± 0.10 [0.20; 0.54] |
| Caecum weight | *g* | 0.02 ± 0.01  [0.001; 0.03] | 0.08 ± 0.02 [0.05; 0.11] | 0.54 ± 0.15 [0.37; 0.82] | 0,55 ± 0.12 [0.41; 0.75] | 0,55 ± 0.09 [0.41; 0.75] | 0.53 ± 0.11 [0.32; 0.70] | 0.71 ± 0.09 [0.60; 0.89] |
| Data is presented as mean ± standard deviation [minimum; maximum] per age group and parameter. BW = body weight. Stomach and caecum were weighed with content.  **n* = 7 | | | | | | | | |
